# A Qualitative Analysis of the Relationship Between Simulated And Clinical Learning Environments in Obstetrics and Gynaecology

**DOI:** 10.1007/s40670-025-02313-y

**Published:** 2025-02-17

**Authors:** Ronan Daly, Daniel Kane, Anne Browne, Karen Flood

**Affiliations:** 1 Department of Obstetrics & Gynaecology, Royal College of Surgeons Ireland, University of Medicine and Health Sciences, 123 St Stephen’s Green, Dublin 2, Ireland; 2 Rotunda Hospital, Parnell Square, Dublin 1, Ireland; 3 School of Medicine, University of Galway, Galway, Ireland

**Keywords:** Simulation, Obstetrics and gynaecology, Labour, Medical education

## Abstract

**Introduction:**

Clinical placement on the labour ward is an essential component of Obstetrics and Gynaecology curricula in medical schools worldwide. This clinical learning environment (CLE) provides students with a formative opportunity for experiential learning around labour and delivery. However, the CLE presents challenges to learning, in particular diversity of experiences and opportunities. The simulated learning environment (SLE) has been adopted by medical schools worldwide in order to address such issues. The SLE provides a safe space for students to practise clinical skills around care in labour and delivery. These learning experiences form the sole exposure for many students to labour and delivery. This study examines the learner experience of these environments and the relationship between them.

**Methods:**

A qualitative research study was performed in the Royal College of Surgeons Ireland. Fourth year undergraduate medical students undertaking their obstetrics and gynaecology rotation were invited to participate. Students attend a labour ward simulation and a week-long clinical placement as part of this rotation. Focus groups were conducted following the simulation and students completed audio diaries during their clinical placement which underwent inductive content analysis.

**Results:**

Four major concepts emerged from analysis of the data from 29 participating students. Simulation was viewed by students as preparation for the CLE. Learner safety within the simulated learning space was highly valued by students. Learner roles in the SLE were often assigned, while student identity on the labour ward developed from their own engagement, patient interactions, and interprofessional staff. Students identified the emotional aspect of the CLE of the labour ward as a significant impact on their learning experience.

**Conclusion:**

The SLE was valued as a stepping-stone to prepare for the CLE. The safety afforded by the simulated labour ward was important to the student learning experience. The CLE was conducive to the formation of learner identities and students valued the emotional engagement with patients. These two areas require further exploration within the simulated learning space.

**Supplementary Information:**

The online version contains supplementary material available at 10.1007/s40670-025-02313-y.

## Introduction

The clinical placement is a formative aspect of medical student education, affording students the opportunity to develop their clinical skills and reasoning (Sellberg, Palmgren, & Möller, 2021). This learning experience encourages higher order learning in medical students by highlighting the complexity of managing illness and working with and patients [36]. The clinical learning environment (CLE) exposes students to the reality of their chosen profession and the art of medicine. Within the context of obstetrics and gynaecology, exposure to labour and delivery is a required component of most curricula. First-hand clinical experience around care in labour and delivery presents an exciting opportunity for medical students to learn about childbirth and care in labour. As a CLE, the labour ward has been shown to consolidate learner knowledge around the care of patients in labour [40]. It affords students the learning opportunity for the application of knowledge and the performance of learned clinical skills [8].

However, the reality of the CLE, while providing students with learning opportunities, can present several challenges. These barriers to learning include competition with other learners, access to patients, and variations in clinical experiences [23, 38]. The reality of interacting with patients can be a source of fear and uncertainty of students [55]. The challenges of the CLE have led medical schools worldwide to adopt simulation in an attempt to overcome them [10]. Simulation is a form of learning instruction which utilises replicated clinical tasks, contexts, and environments to provide a safe environment for learners to develop clinical skills to apply to patient care [13]. The SLE has been shown to allow for a more controlled learning environment for students and a wider variety of experiences [6, 48]. Obstetrics and gynaecology education has readily accepted and incorporated simulation-based teaching that provides a secure atmosphere for students to learn essential skills such as vaginal delivery [13].

Much research has been performed in the area of simulation and obstetrics and gynaecology. This research has primarily focused on the demonstration of improved learner confidence in the labour ward setting and preparedness for practice [9, 21],J. [34, 47]. However, the SLE presents its own barriers to learning. Significant resources and annual financial costs are required for its provision [13]. While the impact of simulation in improving undergraduate knowledge of labour and delivery has been well established [21, 31],J. [34, 39, 41, 46], its effectiveness has not been definitively established in regard to higher order learning such as behavioural change and patient outcomes [11],J. F. [35]. These concepts are underpinned by the concern that exposure to the SLE without corresponding clinical experience may fail to meet the needs of learners [22].

In the context of labour and delivery, both clinical and simulated learning environments are key aspects of medical student learning. The obstetrics and gynaecology rotation may constitute an isolated exposure to learning about labour and delivery for many students as they progress through medical school [35]. The interplay between these two learning environments is therefore of paramount importance to students in obstetrics and gynaecology. This relationship between simulation and the CLE has been explored in the fields of midwifery and postgraduate learning and practice [26, 33]. To our knowledge, the relationship between simulated and clinical labour ward experiences in an undergraduate medical student context has not been explored to date.

This study aimed to explore how simulation and clinical learning interact to better understand the educational role and student experience of these environments. We sought to gain further understanding of the factors affecting learning about labour and delivery in both contexts. Student learning about care in labour and delivery is of considerable importance to the future health of pregnant patients [40]. Investigation into the relationship between these unique learning environments and its impact on the student learning experience can inform practice in this crucial area of medical education. In order to further evaluate the learning experience of students in these contexts, a qualitative examination of simulation and clinical placement was undertaken in the context of labour and delivery.

## Methods

### Research Aim

This longitudinal observational study qualitatively examined simulated and clinical learning environments and their relationship within the context of care in labour and delivery in undergraduate medical education.

### Research Setting

The Royal College of Surgeons Ireland provides a medical degree programme to both undergraduate and postgraduate students over four or five years. As part of the degree programme, students have a mandatory clinical placement in Obstetrics and Gynaecology which is 6 weeks in duration. As part of this, a mandatory week-long labour ward placement is undertaken by fourth year undergraduate medical students at the Royal College of Surgeons Ireland. This clinical placement is preceded by a simulation session encompassing aspects of labour, delivery, and obstetric emergencies. Within 1 week of completion of their simulation session, students are then rostered on three labour ward shifts, where they participate in patient care, work collaboratively with midwifery, obstetric, and anaesthetic staff, and assist at deliveries. New students rotated through their labour ward placement each week during the study period.

### Research Design

We used a longitudinal observation study to answer the research question: “How does the student learning experience in a simulated labour ward environment relate and compare to the in vivo reality?” The qualitative approach was selected in order to capture the complexity of elements which are at play in the clinical and simulated learning environments, and to provide rich data as to the student experience. Focus groups were conducted to provide qualitative data on the simulated learning experience, led by a research assistant. The students would then proceed to attend their clinical labour ward placement and record daily audio diaries reflecting on their learning experience. An exit interview was conducted following completion of the clinical placement to clarify any issues and provide an opportunity for debriefing.

Audio diaries were selected as the primary medium for data collection, providing for reflection and discussion of participant experience in real time [52, 53]. Embedded within a social constructivist paradigm, audio diaries provide for critical reflection of lived experience, through the guidance of a facilitator [20]. Written prompts were provided to participants in facilitate reflection on their learning experiences on the labour ward. Governance and oversight are essential to the use of audio diaries in qualitative research [52], necessitating the employment of elements such as the initial focus groups, written prompts, transcription, and the exit interviews.

This dual method of data collection was adopted in order to give a comprehensive view of the student learning experience in both environments. Focus groups provide for social reflection on the student experience at a fixed-time point [18], creating potential for meaningful and rich data to be collected following completion of their simulated learning experience and orientating students to self-reflection. Conducting audio diaries empowers students to reflect on their identities and share their lived learning experiences in real-time, while providing scope for these experiences to change over time [52].

## Ethics

Ethical approval was granted by the Research Ethics Committee of the Royal College of Surgeons in Ireland prior to commencement of the study (ID: 212,614,454).

## Recruitment and Sampling

For this study, students were invited to participate following completion of their labour ward simulation. Purposeful sampling was utilised in this study in order to provide data based on lived experience relevant to the research question (Benoot, Hannes, & Bilsen, 2016; [37]). Potential participants were identified based on their undertaking of both simulated and clinical labour ward learning environments as mandatory components of their Obstetrics and Gynaecology module. All 144 students attending their Obstetrics and Gynaecology module during the study period were informed of the study by an impartial research assistant. The research assistant had no input or influence relating to student academic standing. Participation was on a voluntary basis. The impartial research representative met with the students again prior to the simulation session and were given a short presentation and participant information leaflet. Informed consent was obtained prior to participation.

## Data Collection

Data were collected through the following methods:

1. A focus group which explored the student experience of simulated labour and delivery. This comprised a 30-min session led by the independent research assistant.
2. Audio diaries submitted during the students’ labour ward placement which explored their experience of the CLE. Diary entries were recorded by the participants and uploaded to secure and pseudonymised online server. Written prompts were provided to students to promote reflection.
3. A semi-structured exit interview which provided for further consideration of the clinical and simulated learning environments and an opportunity for debriefing.

## Data Analysis

All data were transcribed using NVivo Version 12 Pro software. Data underwent inductive content analysis in order to provide for the depth and complexity of data required by the research context (Vears & Gillam, 2022). Two researchers (RD and DK) independently familiarised themselves with the transcribed audio recordings and diaries through repeated readings. Broad content categories relevant to the research question were identified from the data and coded using NVivo software. Data saturation was achieved when no new meaningful codes could be generated from the data, with non-emergence of new codes taken as the point of saturation [43]. The categories arising from the data were similar for both researchers. Iterative re-coding was then conducted independently by both researchers, generating subcategories from the data. These subcategories were discussed and agreed upon by two researchers (RD and DK).

## Results

Twenty-nine students volunteered to participate in the study and participated in the initial focus groups. Seven focus groups were conducted following completion of the simulation session at the commencement of the labour ward placement. Twenty-two audio diaries were submitted by participants detailing their experience of the CLE. Three students participated in the post-placement exit interviews.

Four major content categories emerged from analysis of the transcribed data (Table 1):

1. Simulation viewed by students as preparation for the CLE
2. The importance of safety to student learning
3. Variations in learner identity formation within learning environments
4. Significance of patient relationship to student learning

**Table 1:** Content categories and subcategories

Content category	Subcategory
1. Students view simulated and clinical learning environments as symbiotic	Simulation as stepping stone
	Opportunity for reflection
	Application of knowledge
2. The importance of safety to student learning	Simulation and safety
	Establishing confidence
	Fear of harm
3. Variations in learner identity formation within learning environments	Assigned roles in simulation
	Importance of engagement
	Patient role in identity formation
	Interprofessionalism
4. Significance of emotional response to student learning	Emotional impact of CLE
	Emotion and learning
	Simulation disconnected from reality

Example excerpts from the transcribed data are included in Tables 2, 3, and 4 and Appendix 1.

**Table 2:** Excerpts regarding preparation

Preparation	• Simulation as a stepping-stone to the clinical environment	“A simulated environment was a safe and controlled environment where I had the opportunity to make mistakes, or where anyone has the opportunity to make mistakes and redo, correct my wrongs, practice, right?” “What the simulated environment and managed to do is create a skeleton, so there is a skeleton of what will be done in real life, right? It creates a step-by-step environment and in real life, everyone is doing the same thing simultaneously.” “So the [simulated] experience is a good in, first of all, like preparing us for our first time into a specific clinical setting and then for showing us different scenarios that we might not have seen before. Um, so I really love doing that for obs and gynae, having that kind of [simulated] learning before our week was really helpful.”
	• Opportunity for reflection	“I knew what I was supposed to do, I knew how to handle it. So I feel like, even though, I was on autopilot at that point, I went and helped around with everything that I could, and I guess this is where the simulated environment training that we've had also kind of kicks in because we've had that. So I kind of knew how the worst, *worst* case scenario would be and how to act. So even in this minor scenario, I just thought back to all the training, the simulation that we had and was able to apply it in that sort of way.” “Because technically when I'm reading, I can make all the differentials, I can order all the tests, but in real life, you can't do that. You need to focus on what's more important, on what is more plausible. Of course, like we would put in all the possibilities, but that is kind of like a flow that we need to go through.” “We had time to think. So when the consultant was asking us, “okay, what do you guys think?”, it kind of made our brains move and there was no pressure on time. So I like that where you're kind of facing an actual real life scenario, but at the same time you have the time to think about it and to think about your next move and also seeing how other people react and then maybe changing and learning from that. So I really like being in a group and seeing how everyone would react, and that would give me the opportunity to kind of reflect back on myself and how I'm doing and what things I could incorporate, what things that I could change.”
	• Promotion of critical thinking	“I feel I had the edge over anyone wouldn't have been through the simulation, I feel. If at all, I could have chipped in to help, I would have because I've been through the simulation and I know what's happening now, compared to someone who hasn't went through the simulation.” “But then I initially I couldn't figure out the bearings—like what I was supposed to do first. But then once you started thinking about it, you start flowing in like, OK, I can actually do this, you know?” “But I was confident that I could, you know, do a very small part and what not, you know, not mess it up. And so that would be definitely a huge benefit of what we had seen, what I had seen in the simulated environment. And yeah to know what to expect, what was coming next.” “And I guess it's something I could say I picked off from the simulated environments, much so enough to understand what is going on and why, why everyone is doing what they're doing.”

**Table 3:** Excerpts regarding safety

Safety	• Value of psychological safety	“The way I see it, the simulated environment gets you to hone your skills, do the right thing and be prepared to do the right thing. And then the clinical setting is where everything you need to do there has to be of second nature, and it can only be of second nature if you had the opportunity to practice in a in a safe, controlled environment like a simulated environment. So I feel it's actually a positive feedback sort of environment. The experience you get from a simulated environment, the experience and skill you get from a simulated environment, adds into the clinical skills.” “It's easier to learn, you feel under less stress, and you probably learn better when you're less stressed about it.” “So it was great to have had that first step where everything was broken down into manageable chunks of information and not having the real life stressors in the simulation environment is fantastic. “ “I feel like it’s the ability to make mistakes in a simulation as opposed to if you’re in the real life setting.” “You're here to learn, if you make mistakes, that's a good thing.”
	• Fear of causing harm	“The simulated environment then adds the technique and like the hands on approach, but again keeps you buffered from the kind of overwhelm of a real scenario with all the different people. Like the fact that it's actually a baby and that anything that's going wrong, the stress will go through the roof in the clinical environments. But in the simulated environments, it's all a mannequin and a machine, so it kind of removes a lot of that. So it lets you learn more bite-sized chunks, and then you add the clinical relevance to us where you have the natural, the unpredictable, extreme situations that arise in the clinical setting and the people factors, and the patients, and interacting. So it's nice in that you're incrementally adding and building and building to the real thing and where you have to put all of these things together—the theory, the technical, the patient interaction, the interpersonal interactions, all of it.” “The simulated environment then adds the technique and like the hands on approach, but again keeps you buffered from the kind of overwhelm of a real scenario with all the different people. Like the fact that it's actually a baby and that anything that's going wrong, the stress will go through the roof in the clinical environments. But in the simulated environments, it's all a mannequin and a machine, so it kind of removes a lot of that.”
	• Concerns of students	“The lack of risk that can go wrong is definitely something that like just allows you to learn more because you can do it and you know, at the end of the day, no one's going to die, I suppose.” “So it wasn't again quite as overwhelming with new things coming at me, especially on a night shift when you're a bit kind of disorientated at like three o'clock in the morning.” “And then the clinical setting is an environment of pressure. Time is of the essence, and there's no time to practice whatever you do there. You can't do anything wrong. There are no redos. There are no do overs.”

**Table 4:** Excerpts regarding identity

Identity	• Assigned role in simulation	“With the clinical environment, it's a kind of reality check for us students, it’s that we're used to people assigning as far as telling us what to do, “OK, you do this.”, but now in a clinical environment as part of the team, we have to take the responsibility for that. We have to take the wheel in a way to approach our learning.” “I liked it more when the material that was going to be covered in the simulation cases was given to us beforehand. […] And I feel like that solidifies that more than just background information you have applying it randomly at any moment.”
	• Importance of student engagement	“I tried to help out in any way that I could. The midwife I was with was good in that she asked me to do these things because I felt a bit, you know, I wasn't sure why or whether I should or I shouldn't be doing things. And you obviously, you don't want to feel like you're in the way, but you don't want to feel like you're just a piece of furniture either.” “Just not being like passive. Like actively trying to do something instead of just like listening or something, I think can be really useful, especially when you're doing something like an emergency.” “If you don’t ask questions and you just kind of stand there with your arms folded, like, you’re not going to get anything.” “With the clinical environment, it's a kind of reality check for us students, it’s that we're used to people assigning as far as telling us what to do, “OK, you do this.”, but now in a clinical environment as part of the team, we have to take the responsibility for that. We have to take the wheel in a way to approach our learning.” “Finding a role in a clinical environment is mostly determined by the person themselves, and we shouldn't really wait for another person to tell us what to do.” “Unlike the United States and all those places, we don’t really have a defined role. I feel like we’re kind of just sightseeing, and when like the clock hits 5 or the baby is delivered we kind of just go”
	• Patient factor in identity formation	“The partner was with her, and anything to give the partner a hand with, I was there. I was a front man and I like that. I appreciated how, you know, kind and welcoming that they were for me to do all those things for them. I felt part of the team as well so that helped, that really helped.” “But then I was just sitting around there and she woke up, I just went up and said “Hi, congratulations, you're about to meet your daughter soon, you pulled through”. You know, it was a really surreal experience. I felt part of the journey and it was really nice.” “And I, sort of, just made myself, you know, part of the team and I didn't want to, you know, make myself feel like an outcast a bit. I wouldn't want to be that one guy standing in the corner with, you know, the woman and partner wondering who he is and why is he standing around the corner like that, making it so weird? But then I was involved, I tried to engage with the patient as well, and they as well engaged with me, they reverberated that sort of energy I was giving.”
	• Interprofessionalism in identity formation	“Basically, I feel like the engagement needs to be from both sides for it to be very effective. And I know that's difficult to achieve, but I really do think that if these factors were both right that they could synergize and result in a more positive learning experience. Eagerness from the student and the midwife, basically.” “I could go, speak to the patients and, you know, get as involved as I as I could. Just doing simple jobs like taping the cannula and the blood pressure cuff and stuff like that, they were great that I could do them little small jobs. But that was more defined by the midwife more than anyone.” “The staff really friendly and welcoming, the guys really had open arms for us.”
	• Appetite for interprofessionalism in simulation	“And also the other thing that I think was kind of missing from the simulation was seeing like the interplay between the midwives themselves. So like between the midwife who was in the room and then a more senior midwife who she had to call in to, you know, to ask for her opinion on different, different things at different times and then the different specialties. And so the article, the anaesthetist then obviously to get the epidural and just seeing how the different specialty work kind together and with the patient, something that we didn't necessarily see in these stimulated or simulated environments.” “It's not just going to be one person, you know, if there was other people involved in the simulations, the nurses, midwives, and that would be probably a more realistic simulation for what it's actually like.” “So what the simulated environment fails to capture is that management of obstetric emergencies is multi-disciplinary. So we can't obviously replicate the involvement of the neonatologist and the haematologist and the porter as well. We can't replicate that in a simulated environment. We can only say it—“OK, this is the part where you need to call for help, call for the neonatologist to come in, code for the haematologist for any blood loss and call the porter if you had taken any bloods, call for the other midwives who were on standby.” “In the in the simulation we only had the consultant there. So we only saw it from the point of view of the doctor. But in real life, there are the nurses, there are the midwives, there are so many people involved, the anaesthesiologists. And when they come through [at] different timings, what do they do? How? What are they looking for? Like because every person in the healthcare setting looks for something different. So the midwife would be looking for something a bit different than what the anaesthesiologist would be looking for. So yeah. We don't have a lot of that point of view doing our simulation. But again, it was just like, we did it only once. So maybe if we do it more then we could experience more of those different point of views.”

### Simulation Viewed by Students as Preparation for the CLE, Not a Replacement

Simulation was viewed by students as being a stepping-stone to the CLE, a preparatory learning experience which would provide footing for them in the CLE. Students felt participating in a labour ward simulation helped to apply their existing knowledge and to develop their understanding of the essential elements of the CLE:

You’ve kind of been building incrementally; from the lecture material you learn the theory, then the techniques with the mannequin, and how it’s all put together in real life.

Their labour ward simulation experience provided an opportunity and an impetus to reflect on their existing knowledge through its application:

You go into a room and you realize you’ve no idea where anything is and it’s only then you realize where your big, massive gaps are.

Students valued their learning experiences in the simulated labour ward environment as important to achieving learning in the CLE. The preparation and understanding afforded by the simulated environment allowed students to apply their prior knowledge in the labour ward setting:

It was much less overwhelming when everything was happening fast in a speedy labour when I knew everything from the simulations. […] So when I saw it in real life, it was easier to take in the whole situation around me, instead of being a little bit overwhelmed with everything.

### The Importance of Safety to Student Learning

The concept of safety was a highly prized aspect of the SLE for learners, supporting and facilitating learning around labour and delivery. Several participants noted the importance of practising essential labour ward skills and the management of emergency scenarios in the simulated space, without any potential risk to patients.

I think it's just the whole point is that it also is a very safe space and you can run through all these different scenarios, especially without having like a real patient and having to have a real situation, which is great.

In their simulated learning around labour and delivery, students reported feeling more assured in seizing learning opportunities and participating in the CLE. By removing the fear of causing harm to patients from the learning environment, learners established confidence in their knowledge and ability:

I think as a student, you don't really want to put yourself out there because you don't want to make a mistake that could have a real life impact on people. Whereas now, if I was in the same situation in hospital, and now that I've done this, I would be a lot more confident in my own skills.

The potential for causing harm to patients was an inherent aspect of the CLE noted by several students. This potential was a source of concern which was difficult for some students to balance against their learning.

The clinical setting is an environment of pressure. Time is of the essence, and there's no time to practice whatever you do there. You can't do anything wrong. There are no re-dos. There are no do overs.

### Variations in Learner Identity Formation Within Learning Environments

Their development of a professional identity within the CLE was a complex process dependent on student engagement, patients, and teachers. Participants noted that there was uncertainty as to the extent and meaning of their role within the CLE, unlike the simulated environment where learner roles were assigned by teachers.

In an emergency, you don't get to stand outside the room for five minutes and be like ‘OK, this is what I'll do, this is what you’ll do’, […] you’re just there and you just have to deal with it, with what's in front of you.

Several participants discussed the importance of engagement in creating an identity for themselves within the CLE.

It’s a kind of reality check for us students, it’s that we’re used to people assigning as far as telling us what to do […] But now in a clinical environment, as part of the team, we have to take the responsibility for that. We have to take the wheel, in a way, to approach our learning.

Students valued their interactions with real pregnant people and their birthing companions and considered them to be highly important to their identity and learning in the CLE. Participants did not consider that the SLE was capable of capturing this constructive learning experience.

So I was there with the patient holding her hand and also assisting the registrar. So being involved that deeply with that process was amazing. Like, it showed me what my capabilities are and how to handle these sort of situations, […] all of these things that you can't really replicate in a simulated environment and you only kind of get with real life exposure.

Midwifery, obstetric, and anaesthetic staff were discussed as playing a valuable role in identity formation in the CLE, encouraging student engagement.

[…] the midwives and the Reg are great for actually getting me involved and telling me what to do and showing me what to do […] It did make like I felt like I was a bit more involved and not just in the way that sometimes you do feel in these sort of situations.

Students described a strong appetite for the incorporation of such interprofessional elements into simulation.

In the simulation, we only had the consultant there. So we only saw it from the point of view of the doctor. But in real life, there are the nurses, there are the midwives, there are so many people involved, the anesthesiologists. […] We don't have a lot of that point of view doing our simulation. […] So maybe if we do it more then we could experience more of those different point of views.

### Significance of Emotional Response to Student Learning

Students highlighted the emotional impact of interacting with patients during labour and delivery. The significance of witnessing and participating in a labour and the delivery of a baby was discussed by the students.

You just felt with them, you've just connected to these strangers. You’ve witnessed something amazing in their lives and now you have to say goodbye so it's a bit, kind of, bittersweet in that sense.

Students considered their relationship with patients to be an integral aspect of the CLE, driving learning and development.

It was rewarding to interact with the patients. […] It was shifting me more towards being proactive, doing things independently.

Participation in a labour and involvement in a birth was described as a significantly affecting experience by learners. The profound emotional impact of such a high-pressure clinical environment was noted by the students to be a substantial factor in their learning experience.

Joyful tears. It was… Yeah, it was magical.

It was a bit distressing to me, seeing all that blood, especially to someone who now I know. So it's not just a random patient that I read their file, no, it's someone that I've been talking to for the past three to four hours and now they're bleeding in front of my eyes.

Students criticised the SLE as being disconnected from the emotional resonance of the CLE. Participants felt that simulation was limited in its ability to replicate the human aspect of the learning environment, limiting buy-in by students.

It would be very difficult to simulate the pain that people are in, the emotional intensity of the environment. It’s just a very highly emotional environment.

Authenticity of the SLE was regarded by participants as impacting on their learning. Participants reported a lack of more complex human elements within their simulated learning experience (Table 5).

We will talk to people of different backgrounds, or who have different demeanours, different ways of talking. So in a simulation, it's hard to replicate real human interactions. […] And that's the reality of our work. But yeah, I guess the hardest part to replicate is the human interactions.

**Table 5:** Excerpts regarding emotion

Emotion	• Emotional impact of labour ward	“The gravity of the situation was much more and more intense than in the simulated environment.” “You know, the actual the psychological toll of the labour on the mother, it was something that is hard to simulate as well.” “You know, to my surprise, most of the patients just wanted a surprise baby, they didn't want to know the gender and the magical moment when they find out if it's what they wanted, if they wanted a boy, if they wanted a girl. Joyful tears. It was… Yeah, it was magical.” “It would be very difficult to simulate the pain that people are in, the emotional intensity of the environment. It's just a very highly emotional environment.” “Any aspects of clinical learning environment, which will be difficult to replicate in the simulated environment would be the actual patient's distress and the pain that the patient suffered during the labour. Even with adequate analgesia, it is quite distressful. So I think that just simulating the patient aspect is very difficult.” “And then just seeing the joy in both births, like the joy of the mother finally getting it over with and seeing the baby that they've worked really hard in the past nine months to, like, bring to this world. And yeah, like you just felt with them, you've just connected to these strangers. You’ve witnessed something amazing in their lives and now you have to say goodbye so it's a bit, kind of, bittersweet in that sense, but it was really nice to witness and to see.” “It was a bit distressing to me, seeing all that blood, especially to someone who now I know. So it's not just a random patient that I read their file, no, it's someone that I've been talking to for the past three to four hours and now they're bleeding in front of my eyes. And kind of all of the worst case scenarios are just flashing in my head.”
	• Patient relationship integral to learning	“It is so different to actually support a patient during their delivery and to have a hands-on experience compared to reading lectures or books.” “Because no matter how much we read about every disease and how much you need about medicine, you can't read about patients. You have to be there. You have to interact with patients, their families and like that. That's not something that can be read. It's something that you can experience, in my opinion.” “Like during my labour and delivery, I've seen three extremely different patients, like from how some this was their first pregnancy, some this was their fourth, some were not expecting to get pregnant, but there they are. So it's nice to see how to interact, first of all, with different kind of patients to even learn how to talk to patients. Because as medical students, we're used to talking about these medical issues to each other. So we tend to use more scientific or medical jargon. But then when you talk to a patient, you can't say all of that. They won't understand a thing. So it's kind of like a good practice on how to talk to people and how to kind of simplify our knowledge but still get our points across.” “In fact, a lot of the stuff I saw during the labour ward placement actually did help me in my MCQs where I would use the reasoning that I saw in the labour ward to justify which MCQ option I would pick.” “There’s like a humanity to it, instead of just steps one, two, three.” “And again, like that, we will talk to people of different backgrounds or who have different demeanours, different ways of talking. So kinda in a simulation, it's hard to like, I guess it's hard to replicate real human interactions.”
	• Disconnection of simulation from emotional resonance of clinical environment	“I think it's a great learning tool, obviously, like it won't be exactly like it is in a real clinical scenario, and there definitely is that kind of like divide that you can still feel because you know that it's not real. “ “But even the simulated patient, they're obviously not showing emotion. And like I said, the emotional aspect is missing from the simulation environment.” “Being emotionally present for the patients is something that's not simulated in the environment.” “I think the robot does a good job but, of course, you can’t replace the real person.” “The whole aspect of trying to make everyone in the room as comfortable as possible, the patient, the mother, as well as the partner if there’s a partner. How to talk to the patients continuously and encourage them and those things, I feel like it's hard to simulate those of course.” “The difficult thing to replicate in a simulated environment is kind of the patient interaction, like no matter what in the simulation, you have actors who are instructed to say a certain type of way and react in a certain type of way. And I know they try to make it as realistic as possible and it comes from actual interactions with people, but like I would personally know that this is acting. The person in front of me knows that this is acting, so you don't really get into the reality of it in a way, I guess.”

## Discussion

*“Simulated birth and real-world birth are two very different things.”*

### Students View Simulated and Clinical Learning Environments as Symbiotic

Simulated learning environments have been readily assimilated into teaching within the field of obstetrics and gynaecology, with its beginnings being found in the instruction of undergraduate medical students [49]. Simulation is employed in teaching across a wide range of topics essential to learning in obstetrics and gynaecology, from communication skills to pelvic examination to vaginal delivery [13]. Within the sphere of midwifery education, a dialogue exists regarding the replacement of the CLE with simulation [7, 48]. Students in the present study found that the SLE to be a preparatory exercise for the CLE, rather than a substitute. Simulation has been demonstrated to result in greater preparedness to participate in a delivery, increased levels of understanding, and improved student confidence [31],J. [34, 47]. Simulation is effective in preparing students for the technical aspects of a vaginal delivery, as well as providing a valuable opportunity for feedback [31]. Having engaged with the SLE, participants in our study described feeling more confident and having a deeper understanding of labour and delivery upon entering their clinical placements. Within midwifery education, the simulated labour ward has not been found to meet the needs of learners in regard to communication with patients and application of knowledge to the same extent as the CLE [27]. Our study showed that students similarly value simulation as a preparatory learning experience for the labour ward, but not its replacement. The labour ward as a CLE is perceived by students to be an important component of their learning, providing for application of knowledge and performance of clinical skills [8].

### The Importance of Safety to Student Learning

Students in the study at hand perceived the SLE as one of safety, highly valuing this aspect of the learning environment. The security provided by simulation in education is one of its most prized features, fostering a safe space where learning, confidence, and clinical skills can be developed (Andreatta, Bullough, & Marzano, 2010). Unlike the CLE, the SLE empowers students to take ownership of their errors and mistakes, enabling them to be experienced deliberately and used as opportunities for learning [22]. Simulation in medical education is underpinned by this concept, allowing for safe practice of clinical skills without risking harm for the patient [6]. The potential for causing harm to real patients through the authentic interactions of the CLE was highlighted in the present study as a source of concern and anxiety for students. This fear of causing harm has been noted as having a significant impact on the learner during clinical placement [45].

Such fears contrast to the safety of the SLE, where the safety of students themselves is prioritised. The concept of safety is foundational to simulation, allowing students to engage with clinical scenarios without fear of judgement or ridicule, and enable the transformation of practice into learning [42]. Participants reported that the SLE allows for mistakes to become learning experiences and points of growth for their development. Facilitators and teachers within the simulated learning space can foster a safe learning environment through pre-briefing, debriefing, appropriate feedback, and a commitment to respecting learners [30]. The CLE has been criticised for not similarly prioritising learner safety through fears of inadequacy, a hierarchical organisational structure, and belittlement of learners, although these factors are not ubiquitous to all clinical placements [3].

### Variations in Learner Identity Formation Within Learning Environments

Participants highlighted their roles and identities within clinical and simulated learning environments as being an important aspect of their learning experiences. Students reported that their identities within the CLE were developed from a number of sources, including their own engagement and motivation, interactions with patients, and interactions with other professionals. The establishment of a learner identity is a process which evolves from personal and socio-cultural development of the learner [36]. The CLE provides fertile ground for this development. Through undertaking such a placement, the professional identity of students can be shaped through first-hand experience of patient interactions, interactions with other healthcare workers, collaborations with role models, and organisational structures [54]. Students reported that their relationships with patients and staff were instrumental to understanding where they fit in within the labour ward environment. Such contributions to patient care and interactions with healthcare professionals are essential elements of the CLE which contribute to the formation of both clinical competence and the construction of identity [12]. While there is a paucity of studies investigating identity within the labour ward learning environment, such emotive situations can create a crystallised opportunity for the development of professional identity, when adequately supported by teachers [28]. The labour ward is a multidisciplinary clinical environment, with the interactions between midwifery, obstetric, and anaesthetic staff having a significant bearing on the student experience [8]. Participants found that the labour ward provided them with learning opportunities from a range of teachers across medical specialities.

Simulation has been posited as a potential space within which the professional identity of students can be advanced. Initial evidence suggests that by allowing learners to think, act, and feel like professionals, the SLE may help to expedite the maturation of a professional identity (Tien, Wyatt, Tews, & Kleinheksel, 2019). In contrast to the CLE, where identities developed from personal, contextual, and interpersonal experience, participants in our study found difficulty with the allocation of such identities and roles in simulation. Learner roles within the SLE are often designated to students, with students being given their identities within a scenario randomly or by assignment [1]. The development of identity through simulation requires careful balance between authenticity of experience and engulfing students with tasks so as to prevent reflective practice [14]. The designation of roles to learners within the SLE has been shown to promote participation and engagement [5]; however, its impact on professional identity formation is as yet unknown. Learners in our study highlighted the assignment of their identities and roles within the simulated space, while the CLE allowed for more intuitive and self-directed growth in this regard.

### Significance of Emotional Response to Student Learning

Participants reported the emotional aspects of the CLE of the labour ward as having a significant bearing on their learning experience. Emotions experienced in learning environments have a significant influence on student learning through the facilitation of cognition, memory, attention, motivation, and clinical judgment [25]. The relationship between emotion and learning is complex. Both positive and negative emotions can create opportunities for learning, dependent on the nature of the emotion, the learning environment, and the emotional state of the learner [24, 32]. The emotional responses elicited in the CLE relate to student experiences of confronting patient suffering and illness, difficult interpersonal relationships, and clinical dilemmas [28]. Students in the present study noted the complexity of the labour ward as such an environment and found it to be an involving and engaging experience. Such engagement with the CLE has been demonstrated to impact positively on student motivation to learn [19]. The CLE of the labour ward is an especially emotionally diverse environment, with the potential for a wide range of emotions, feelings, and outcomes. The participants in our study reported on the diverse emotional experiences of their labour ward placement, from poignant joy to fear and sorrow, reflective of the human experience of labour and birth. The heavy emotional load created by first-hand experience of a labour and birth is an affecting and formative experience for many students [40].

Our study demonstrated that students considered the emotions of the CLE to be an integral aspect of their learning experience, while they found the SLE did not sufficiently replicate the poignancy of labour and birth. One of the factors which can promote or suppress learning within the SLE is the emotional realism and engagement it creates for learners [29]. The emotional response engendered by simulation is a distinctive one, discrete from the CLE. It is derived from both the content of the scenario, the context of the simulation, and relevant academic concerns regarding achievement and performance [17]. Research on emotional aspects of simulated learning has highlighted the connection to the cognitive load placed on students. Emotions experienced by learners during simulation have been suggested to be facilitative to learning until a certain saturation point, and then can become detrimental to learning [15, 16, 51]. Emotions are inherent to the labour ward as a CLE due to its nature; however, the optimal level of emotion created in the SLE within the context of labour and delivery is not clear.

## Limitations and Strengths

The primary limitation of the study was the high rate of attrition. Twenty-nine students participated in the initial focus groups, while three completed the exit interviews. This attrition rate is likely due to the exit interviews being held following completion of the rotation in obstetrics and gynaecology. Student motivation to continue to participate was likely impacted by time constraints and academic demands of commencing a new clinical rotation. This highlights the importance of timing of exit interviews in utilising audio diaries as a medium of data collection. Participants were selected by purposeful sampling and all were undergraduate medical students from a single medical school and a single academic year. While the overall numbers involved in the study were small, they did reflect a diverse clinical learning experience as students undertook their placements across a range of maternity units (from large tertiary care centres to smaller peripheral units). As teaching staff were involved in the study, the use of an independent research assistant as a gate-keeper was employed to negate concerns regarding confidentiality and academic standing. As participants shared both positive and negative aspects of their learning experiences, this may represent a confidence in the study methodology and a strength of the study itself. Two researchers (RD and DK) conducted the analysis of the data, enhancing the rigour of the study.

## Conclusion

Clinical and simulated learning environments are instrumental to student learning around labour and delivery in undergraduate medical education. The relationship between clinical placement and simulation is a symbiotic one, with both environments having the potential to be informed and transformed by the other. Simulation does not constitute a replacement for the CLE, instead forming an important stepping-stone to the CLE. This study highlights the dialogue created between these distinct environments within the learning process and how they can be reflective and contrasting of each other. There is potential for the CLE to be informed by essential aspects of simulation, such as safety. Correspondingly, the formation of learner identity and the incorporation of emotional learning are aspects of the CLE which could be further explored through simulation.

## Supplementary Information

Below is the link to the electronic supplementary material.

Supplementary file1 (DOCX 31 KB)
