# Supplementary material for: A Qualitative Analysis of the Relationship Between Simulated And Clinical Learning Environments in Obstetrics and Gynaecology
Source: Med Sci Educ. 2025 Feb 17;35(3):1371–83. doi: 10.1007/s40670-025-02313-y (PMC12228605; doi:10.1007/s40670-025-02313-y)
Supplement: Supplementary file 1 — Supplementary file1 (DOCX 31 KB) [file 40670_2025_2313_MOESM1_ESM.docx]

**Supplementary Information**

**Appendix 1:**

**Table:** Excerpts exploring clinical and simulated learning environments.

| **Concept:** | **Subcategory:** | **Examples:** |
| --- | --- | --- |
| **Preparation** | - Simulation as a stepping-stone to the clinical environment | “A simulated environment was a safe and controlled environment where I had the opportunity to make mistakes, or where anyone has the opportunity to make mistakes and redo, correct my wrongs, practice, right?”  “What the simulated environment and managed to do is create a skeleton, so there is a skeleton of what will be done in real life, right? It creates a step-by-step environment and in real life, everyone is doing the same thing simultaneously.”  “So the [simulated] experience is a good in, first of all, like preparing us for our first time into a specific clinical setting and then for showing us different scenarios that we might not have seen before. Um, so I really love doing that for obs and gynae, having that kind of [simulated] learning before our week was really helpful.” |
|  | - Opportunity for reflection | “I knew what I was supposed to do, I knew how to handle it. So I feel like, even though, I was on autopilot at that point, I went and helped around with everything that I could, and I guess this is where the simulated environment training that we've had also kind of kicks in because we've had that. So I kind of knew how the worst, *worst* case scenario would be and how to act. So even in this minor scenario, I just thought back to all the training, the simulation that we had and was able to apply it in that sort of way.”  “Because technically when I'm reading, I can make all the differentials, I can order all the tests, but in real life, you can't do that. You need to focus on what's more important, on what is more plausible. Of course, like we would put in all the possibilities, but that is kind of like a flow that we need to go through.”  “We had time to think. So when the consultant was asking us, “okay, what do you guys think?”, it kind of made our brains move and there was no pressure on time. So I like that where you're kind of facing an actual real life scenario, but at the same time you have the time to think about it and to think about your next move and also seeing how other people react and then maybe changing and learning from that. So I really like being in a group and seeing how everyone would react, and that would give me the opportunity to kind of reflect back on myself and how I'm doing and what things I could incorporate, what things that I could change.” |
|  | - Promotion of critical thinking | “I feel I had the edge over anyone wouldn't have been through the simulation, I feel. If at all, I could have chipped in to help, I would have because I've been through the simulation and I know what's happening now, compared to someone who hasn't went through the simulation.”  “But then I initially I couldn't figure out the bearings - like what I was supposed to do first. But then once you started thinking about it, you start flowing in like, OK, I can actually do this, you know?”  “But I was confident that I could, you know, do a very small part and what not, you know, not mess it up. And so that would be definitely a huge benefit of what we had seen, what I had seen in the simulated environment. And yeah to know what to expect, what was coming next.”  “And I guess it's something I could say I picked off from the simulated environments, much so enough to understand what is going on and why, why everyone is doing what they're doing.” |
| **Safety** | - Value of psychological safety | “The way I see it, the simulated environment gets you to hone your skills, do the right thing and be prepared to do the right thing. And then the clinical setting is where everything you need to do there has to be of second nature, and it can only be of second nature if you had the opportunity to practice in a in a safe, controlled environment like a simulated environment. So I feel it's actually a positive feedback sort of environment. The experience you get from a simulated environment, the experience and skill you get from a simulated environment, adds into the clinical skills.”  “It's easier to learn, you feel under less stress, and you probably learn better when you're less stressed about it.”  “So it was great to have had that first step where everything was broken down into manageable chunks of information and not having the real life stressors in the simulation environment is fantastic. “  “I feel like it’s the ability to make mistakes in a simulation as opposed to if you’re in the real life setting.”  “You're here to learn, if you make mistakes, that's a good thing.” |
|  | - Fear of causing harm | “The simulated environment then adds the technique and like the hands on approach, but again keeps you buffered from the kind of overwhelm of a real scenario with all the different people. Like the fact that it's actually a baby and that anything that's going wrong, the stress will go through the roof in the clinical environments. But in the simulated environments, it's all a mannequin and a machine, so it kind of removes a lot of that. So it lets you learn more bite-sized chunks, and then you add the clinical relevance to us where you have the natural, the unpredictable, extreme situations that arise in the clinical setting and the people factors, and the patients, and interacting. So it's nice in that you're incrementally adding and building and building to the real thing and where you have to put all of these things together - the theory, the technical, the patient interaction, the interpersonal interactions, all of it.”  “The simulated environment then adds the technique and like the hands on approach, but again keeps you buffered from the kind of overwhelm of a real scenario with all the different people. Like the fact that it's actually a baby and that anything that's going wrong, the stress will go through the roof in the clinical environments. But in the simulated environments, it's all a mannequin and a machine, so it kind of removes a lot of that.” |
|  | - Concerns of students | “The lack of risk that can go wrong is definitely something that like just allows you to learn more because you can do it and you know, at the end of the day, no one's going to die, I suppose.”  “So it wasn't again quite as overwhelming with new things coming at me, especially on a night shift when you're a bit kind of disorientated at like three o'clock in the morning.”  “And then the clinical setting is an environment of pressure. Time is of the essence, and there's no time to practice whatever you do there. You can't do anything wrong. There are no redos. There are no do overs.” |
| **Identity** | - Assigned role in simulation | “With the clinical environment, it's a kind of reality check for us students, it’s that we're used to people assigning as far as telling us what to do, “OK, you do this.”, but now in a clinical environment as part of the team, we have to take the responsibility for that. We have to take the wheel in a way to approach our learning.”  “I liked it more when the material that was going to be covered in the simulation cases was given to us beforehand. […] And I feel like that solidifies that more than just background information you have applying it randomly at any moment.” |
|  | - Importance of student engagement | “I tried to help out in any way that I could. The midwife I was with was good in that she asked me to do these things because I felt a bit, you know, I wasn't sure why or whether I should or I shouldn't be doing things. And you obviously, you don't want to feel like you're in the way, but you don't want to feel like you're just a piece of furniture either.”  “Just not being like passive. Like actively trying to do something instead of just like listening or something, I think can be really useful, especially when you're doing something like an emergency.”  “If you don’t ask questions and you just kind of stand there with your arms folded, like, you’re not going to get anything.”  “With the clinical environment, it's a kind of reality check for us students, it’s that we're used to people assigning as far as telling us what to do, “OK, you do this.”, but now in a clinical environment as part of the team, we have to take the responsibility for that. We have to take the wheel in a way to approach our learning.”  “Finding a role in a clinical environment is mostly determined by the person themselves, and we shouldn't really wait for another person to tell us what to do.”  “Unlike the United States and all those places, we don’t really have a defined role. I feel like we’re kind of just sightseeing, and when like the clock hits 5 or the baby is delivered we kind of just go” |
|  | - Patient factor in identity formation | “The partner was with her, and anything to give the partner a hand with, I was there. I was a front man and I like that. I appreciated how, you know, kind and welcoming that they were for me to do all those things for them. I felt part of the team as well so that helped, that really helped.”  “But then I was just sitting around there and she woke up, I just went up and said “Hi, congratulations, you're about to meet your daughter soon, you pulled through”. You know, it was a really surreal experience. I felt part of the journey and it was really nice.”  “And I, sort of, just made myself, you know, part of the team and I didn't want to, you know, make myself feel like an outcast a bit. I wouldn't want to be that one guy standing in the corner with, you know, the woman and partner wondering who he is and why is he standing around the corner like that, making it so weird? But then I was involved, I tried to engage with the patient as well, and they as well engaged with me, they reverberated that sort of energy I was giving.” |
|  | - Interprofessionalism in identity formation | “Basically, I feel like the engagement needs to be from both sides for it to be very effective. And I know that's difficult to achieve, but I really do think that if these factors were both right that they could synergize and result in a more positive learning experience. Eagerness from the student and the midwife, basically.”  “I could go, speak to the patients and, you know, get as involved as I as I could. Just doing simple jobs like taping the cannula and the blood pressure cuff and stuff like that, they were great that I could do them little small jobs. But that was more defined by the midwife more than anyone.”  “The staff really friendly and welcoming, the guys really had open arms for us.” |
|  | - Appetite for interprofessionalism in simulation | “And also the other thing that I think was kind of missing from the simulation was seeing like the interplay between the midwives themselves. So like between the midwife who was in the room and then a more senior midwife who she had to call in to, you know, to ask for her opinion on different, different things at different times and then the different specialties. And so the article, the anaesthetist then obviously to get the epidural and just seeing how the different specialty work kind together and with the patient, something that we didn't necessarily see in these stimulated or simulated environments.”  “It's not just going to be one person, you know, if there was other people involved in the simulations, the nurses, midwives, and that would be probably a more realistic simulation for what it's actually like.”  “So what the simulated environment fails to capture is that management of obstetric emergencies is multi-disciplinary. So we can't obviously replicate the involvement of the neonatologist and the haematologist and the porter as well. We can't replicate that in a simulated environment. We can only say it - “OK, this is the part where you need to call for help, call for the neonatologist to come in, code for the haematologist for any blood loss and call the porter if you had taken any bloods, call for the other midwives who were on standby.”  “In the in the simulation we only had the consultant there. So we only saw it from the point of view of the doctor. But in real life, there are the nurses, there are the midwives, there are so many people involved, the anaesthesiologists. And when they come through [at] different timings, what do they do? How? What are they looking for? Like because every person in the healthcare setting looks for something different. So the midwife would be looking for something a bit different than what the anaesthesiologist would be looking for. So yeah. We don't have a lot of that point of view doing our simulation. But again, it was just like, we did it only once. So maybe if we do it more then we could experience more of those different point of views.” |
| **Emotion** | - Emotional impact of labour ward | “The gravity of the situation was much more and more intense than in the simulated environment.”  “You know, the actual the psychological toll of the labour on the mother, it was something that is hard to simulate as well.”  “You know, to my surprise, most of the patients just wanted a surprise baby, they didn't want to know the gender and the magical moment when they find out if it's what they wanted, if they wanted a boy, if they wanted a girl. Joyful tears. It was… Yeah, it was magical.”  “It would be very difficult to simulate the pain that people are in, the emotional intensity of the environment. It's just a very highly emotional environment.”  “Any aspects of clinical learning environment, which will be difficult to replicate in the simulated environment would be the actual patient's distress and the pain that the patient suffered during the labour. Even with adequate analgesia, it is quite distressful. So I think that just simulating the patient aspect is very difficult.”  “And then just seeing the joy in both births, like the joy of the mother finally getting it over with and seeing the baby that they've worked really hard in the past nine months to, like, bring to this world. And yeah, like you just felt with them, you've just connected to these strangers. You’ve witnessed something amazing in their lives and now you have to say goodbye so it's a bit, kind of, bittersweet in that sense, but it was really nice to witness and to see.”  “It was a bit distressing to me, seeing all that blood, especially to someone who now I know. So it's not just a random patient that I read their file, no, it's someone that I've been talking to for the past three to four hours and now they're bleeding in front of my eyes. And kind of all of the worst case scenarios are just flashing in my head.” |
|  | - Patient relationship integral to learning | “It is so different to actually support a patient during their delivery and to have a hands-on experience compared to reading lectures or books.”  “Because no matter how much we read about every disease and how much you need about medicine, you can't read about patients. You have to be there. You have to interact with patients, their families and like that. That's not something that can be read. It's something that you can experience, in my opinion.”  “Like during my labour and delivery, I've seen three extremely different patients, like from how some this was their first pregnancy, some this was their fourth, some were not expecting to get pregnant, but there they are. So it's nice to see how to interact, first of all, with different kind of patients to even learn how to talk to patients. Because as medical students, we're used to talking about these medical issues to each other. So we tend to use more scientific or medical jargon. But then when you talk to a patient, you can't say all of that. They won't understand a thing. So it's kind of like a good practice on how to talk to people and how to kind of simplify our knowledge but still get our points across.”  “In fact, a lot of the stuff I saw during the labour ward placement actually did help me in my MCQs where I would use the reasoning that I saw in the labour ward to justify which MCQ option I would pick.”  “There’s like a humanity to it, instead of just steps one, two, three.”  “And again, like that, we will talk to people of different backgrounds or who have different demeanours, different ways of talking. So kinda in a simulation, it's hard to like, I guess it's hard to replicate real human interactions.” |
|  | - Disconnection of simulation from emotional resonance of clinical environment | “I think it's a great learning tool, obviously, like it won't be exactly like it is in a real clinical scenario, and there definitely is that kind of like divide that you can still feel because you know that it's not real. “  “But even the simulated patient, they're obviously not showing emotion. And like I said, the emotional aspect is missing from the simulation environment.”  “Being emotionally present for the patients is something that's not simulated in the environment.”  “I think the robot does a good job but, of course, you can’t replace the real person.”  “The whole aspect of trying to make everyone in the room as comfortable as possible, the patient, the mother, as well as the partner if there’s a partner. How to talk to the patients continuously and encourage them and those things, I feel like it's hard to simulate those of course.”  “The difficult thing to replicate in a simulated environment is kind of the patient interaction, like no matter what in the simulation, you have actors who are instructed to say a certain type of way and react in a certain type of way. And I know they try to make it as realistic as possible and it comes from actual interactions with people, but like I would personally know that this is acting. The person in front of me knows that this is acting, so you don't really get into the reality of it in a way, I guess.” |

**Appendix 2**

**Example topics for Semi-Structured interviews**

1. **Post Simulated Learning Environment on Labour and Delivery:**

**We would like to understand more about how students learn in a simulated learning environment.**

**Before this session, did you have much experience of simulated learning?**

- Conversational opening question to put students at ease and encourage reflection.
- Example probing questions: What appeals to you about simulated teaching sessions? Are there things about simulation that don’t appeal to you? What do you hope to gain or learn from a simulated session?

**Regarding the session today, what were your impressions of simulation in this context?**

- Example probing questions: Did it feel authentic? Did you think it was relevant to your learning around labour and delivery? What aspects did you find worked well/not so well?

**What did you think you brought to the session?**

- Example probing questions: Did your prior knowledge prepare you for the session? Do you feel the simulation helped to identify gaps in your knowledge? Did you feel you had a role within the session?

**What do you think the session brought to your learning?**

- Example probing questions: Do you think simulation helped to add to your knowledge about the process and potential problems of labour? Did you feel it gave you an opportunity to apply your knowledge? What did you learn about meeting a patient’s needs? Did you have an opportunity for feedback?

1. **Post Clinical Learning Environment on Labour and Delivery**

**We would like to understand more about how students learn in a simulated learning environment.**

**Before this week, did you have much experience of clinical or workplace learning?**

- Example probing questions: What appeals to you about clinical placement? Are there things about placement that don’t appeal to you? What do you hope to gain or learn from a clinical learning session?

**Regarding the session today, what were your impressions of clinical learning in this context?**

- Example probing questions: Did you think it was relevant to your learning around labour and delivery? What aspects did you find worked well/not so well? Were the learning outcomes clear?

**What did you think you brought to the clinical learning environment?**

- Example probing questions: Did your prior knowledge prepare you for the session? Do you feel the placement helped to identify gaps in your knowledge? Did the simulation session help you to prepare? Was there anything missing from your prior knowledge? Did you feel you had a role within the workplace?

**What do you think the session brought to your learning?**

- Example probing questions: Do you think clinical placement helped to add to your knowledge about the process and potential problems of labour? What did you learn about meeting a patient’s needs? Did you have an opportunity for feedback?

**What are your thoughts about your simulation learning having completed your clinical placement?**

- Example probing questions: What differences did you notice? What similarities between the two environments did you notice? Did you feel the clinical learning environment gave you an opportunity to apply your knowledge?

**Appendix 3**

**Audio Diary Prompts**

**Audio Diary Example Prompts**

The purpose of such prompts are to encourage reflection.

Examples would include:

- What aspects of the clinical environment did you find helpful to your learning?
- Were there any barriers to your learning in the clinical environment?
- Do you feel you had a defined role within your placement in relation to other healthcare professionals?
- What was your experience of interacting with patients?
- Do you feel the simulated environment prepared you for the clinical environment?
- Were any aspects of the clinical environment explored in the simulated environment?
- Were there any aspects of the clinical learning environment which would be difficult to replicate in the simulated environment?
- Were there any similarities between the clinical and simulated learning environments?
- How do you feel the different environments impacted on your learning?
